# Revisiting Glutathione Transferases of *Plasmodium* as Potential Targets for Coadjuvant Therapies and the Development of New Antimalarials

**DOI:** 10.3390/ph19081287

**Published:** 2026-08-14

**Authors:** Maria Luiza Lima, Nele Wild, Eva Liebau, Carsten Wrenger, André Barateiro

**Affiliations:** 1 Department of Parasitology, Institute of Biomedical Sciences, University of São Paulo, Av. Prof. Lineu Prestes 1374, São Paulo 05508-000, Brazil; marialuizalima@usp.br (M.L.L.); nwild@usp.br (N.W.); 2 Institute of Integrated Cell Biology and Physiology, University of Münster, Schlossplatz 8, D-48143 Münster, Germany; liebaue@uni-muenster.de; 3 Institut Pasteur de São Paulo, Av. Prof. Lúcio Martins Rodrigues 370, São Paulo 05508-020, Brazil; 4 Center for Tropical and Emerging Global Diseases, University of Georgia, 500 D. W. Brooks Drive, Athens, GA 30605, USA

**Keywords:** malaria, *Plasmodium*, redox homeostasis, glutathione S-transferase, drug discovery, target candidate profiling

## Abstract

Malaria remains a major global health problem, especially in low- and mid-income countries where *Plasmodium* parasites are commonly endemic. Despite the significant number of available drugs to treat the disease, some aspects, such as increasing resistance to frontline antimalarials, inefficient treatment of asymptomatic carriers and treatment inadequacy for certain vulnerable groups, are jeopardizing control measures, justifying the need for therapeutic targets driving the discovery of new compounds. In this context, glutathione transferases, which are essential for redox metabolism and detoxification of heme in the asexual blood stages of *Plasmodium* parasites, have emerged as promising targets more than two decades ago, but have been deprioritized due to significant gaps in research. This review aims to synthesize current biochemical, structural, genetic, and transcriptomic evidence on *Plasmodium* glutathione S-transferases and evaluate their role in parasite physiology across the life cycle while revising their potential for drug targeting using suggested guidelines for target candidate profiling and prioritization.

## 1. Introduction

Malaria still remains one of the biggest global health challenges, affecting mostly people in low- and mid-income countries. It is an infectious vector-borne disease caused by protozoan parasites of the genus *Plasmodium*, which are spread by infected females of the *Anopheles* mosquito and represent a significant source of morbidity and mortality for vulnerable populations worldwide. Despite the significant diversity of malaria parasites causing disease in humans, two species in particular—*Plasmodium falciparum* (*P. falciparum*) and *Plasmodium vivax* (*P. vivax*)—have historically been the most clinically relevant species due to their contribution to the global burden of malaria and their intrinsic species-specific features that impose significant challenges to the eradication agenda [1,2].

Part of the deployed control measures have often consisted of treating parasite carriers with antimalarials, a strategy that, over the years, has been critical for controlling cases and preventing deaths around the world. Unfortunately, progress has stalled and we are now unlikely to meet the goals defined in the Global Technical Strategy 2016–2030, aiming to reduce malaria incidence and mortality by 90% until 2030 [3]; this drawback is largely a consequence of treatment inadequacy promoted by the onset of drug-resistant parasites, the incapacity to efficiently treat cryptic stages in asymptomatic carriers, as well as the lack of safety and tolerance of particular vulnerable groups to available compounds (e.g., pediatric patients and pregnant women) [1].

The primary challenge of treating malaria caused by *P. falciparum* is its widespread resistance to virtually all available antimalarials [4,5]. The current frontline treatment recommended by the World Health Organization is artemisinin-based combination therapies (ACT) [2], consisting of a potent fast-acting artemisinin derivative and a longer-lasting partner drug that ensures clearance of residual parasites over time [1]. The first evidence of artemisinin treatment failure came from the Greater Mekong Region in 2008 [6] and was afterward linked to the onset of *pfkelch13* mutants [4,5], which are now highly prevalent across endemic areas and may still contribute to over 100 000 estimated additional deaths every year if left unchecked [7]. Likewise, additional mutations in genes such as *pfcrt*, *pfmdr1*, *pfcytb* and *pfdhfr* have been critical in driving resistance to partner drugs such as mefloquine, sulphadoxine, piperaquine and lumefantrine, as well as other compounds such as quinine, chloroquine, atovaquone and amodiaquine, which were once efficient as a monotherapy treatment [4,5]. *P. vivax*, on the other hand, is still without a significant number of resistance-associated polymorphisms detected [4,5]; difficulties in eliminating this species are often associated with a reduced capacity to identify asymptomatic carriers harboring low-density infections and dormant hypnozoites in the liver and limitations in performing mass drug administration of primaquine and tafenoquine, which cannot be prescribed to pregnant women and require a diagnostic of proper glucose-6-phosphate dehydrogenase activity before administration to adults [1,8].

To stay on track with the eradication agenda, newly improved compounds are required to face existing and emerging challenges. In this context, Novartis has recently announced the successful phase III clinical trial of ganaplacide–lumefratrine (GanLum) as an alternative to artemether–lumefantrine (Coartem) to treat acute and uncomplicated malaria while overcoming the growing threat of resistant parasites [9]. These findings are uplifting, yet we know that emerging resistance has been constant throughout history and therefore inevitable. As such, there is a continuous need for the identification of new targets in *Plasmodium* parasites that justify the development of improved molecules for new antimalarial therapies [10]. Herein, we provide an updated and comprehensive overview of the structural, biochemical, and functional characteristics of *Plasmodium* glutathione S-transferase (GST) while revisiting its potential as a therapeutic target. Despite early promises, the GST of *Plasmodium* parasites has received limited attention and has been largely underexplored over the past years, underscoring the need for a renewed and detailed evaluation of its potential as a drug target for the development of novel antimalarials.

## 2. Hemoglobin Metabolism and Oxidative Stress Responses in *Plasmodium* Parasites

The *Plasmodium* complex life cycle involves two hosts and multiple development stages, which forces the parasite to adjust its metabolism in response to changes in temperature, pH, oxygen levels and available nutrients. In particular, during the intraerythrocytic development, the parasite undergoes rapid growth and multiplication, resulting in an elevated metabolic rate that drives the continuous production of reactive oxygen species (ROS) through both parasite- and host-dependent processes. A typical example is the hemoglobin degradation that occurs within the parasite’s acidic food vacuole; this critical process supplies amino acids to the parasite, but concomitantly generates redox-active by-products, including toxic free heme (ferri/ferro protoporphyrin IX, FP) and hydrogen peroxide (H_2_O_2_). The majority of FP is neutralized, either by conversion to crystalline hemozoin or via alternative detoxification mechanisms involving reactions with glutathione or FP-binding proteins. However, any unneutralized FP can cause redox damage to host proteins, disrupt parasite enzymes, and rupture erythrocytes [11]. Although most free heme is converted into hemozoin, some of it, still in its ferrous state (Fe^2+^), can be reduced in two different ways: 1) in contact with oxygen (O_2_), leading to the generation of superoxide (O_2_^−^) and hydrogen peroxide (H_2_O_2_), or 2) in direct contact with H_2_O_2_, forming other ROS such as hydroxyl radical (·OH), hydroxide (OH^−^) and ferric iron (Fe^3+^), with intrinsic oxidative potential, in a process known as the Fenton reaction [11,12]. This exposure to oxidative stress is also exacerbated by the parasite’s rapid metabolism and ROS produced by the host’s immune system [11]. ROS causes significant oxidative damage to DNA, protein misfolding and aggregation, and lipid peroxidation, ultimately resulting in irreversible membrane injury and cell death [11,13,14]. Therefore, ROS overproduction is a key mechanism exploited by many conventional antimalarial drugs, including artemisinin derivatives, atovaquone and quinolones, which either directly or indirectly generate ROS to kill the parasite [12].

The main players in the redox balance are the low molecular weight thiol/disulfide pairs such as reduced glutathione (GSH) and its oxidized form (GSSG), whose levels are regulated by synthesis, reduction and efflux. Functionally, glutathione exerts its protective effects primarily as reduced GSH, and any oxidized GSSG is restored to sustain the usually high ratio of GSH to GSSG and thus the cellular redox balance. These protective effects are catalyzed by enzymes such as the glutathione S-transferases (GSTs), which use GSH as a cofactor during antioxidative and detoxification reactions [11,13,14].

## 3. Glutathione S-Transferases in Humans and Malaria Parasites: Same but Different

### 3.1. The Superfamily of Glutathione S-Transferases and Human Isoforms

GSTs (EC 2.5.1.18) are a superfamily of multifunctional enzymes, located in membranes or cytosol, found in a multitude of organisms across widely unrelated taxonomic groups. First isolated from the cytoplasmic fraction of rat liver tissue in the early 1960s [15], GSTs have since been identified in diverse organisms, from prokaryotes to mammals; their evolutionary conservation underscores their fundamental role in cellular adaptive mechanisms, especially those involved in stress responses. More specifically, these enzymes catalyze the nucleophilic addition of reduced GSH to a wide range of endogenous and exogenous electrophilic and usually toxic molecules, enhancing their solubility and thereby facilitating excretion. Moreover, due to their versatility, the functional repertoire of GSTs extends beyond their canonical role, as some isoforms in this superfamily exhibit secondary enzymatic activity as isomerases (e.g., prostaglandin synthesis), peroxidases (e.g., reduction of lipid peroxidation), thiol transferases, among others, as well as non-enzymatic activity as transducers of signaling pathways by directly interacting with kinases [16,17,18,19]. This functional diversity positions GSTs as critical players in broader metabolic, inflammation, and cell signaling regulation.

However, GSTs are better known for their ability to use GSH to detoxify hydrophobic xenobiotics (e.g., pesticides, herbicides, and antibiotics) and also endogenous electrophiles generated during oxidative stress. Additionally, in line with their cytoprotective role, these enzymes exhibit remarkable ligand-binding versatility, acting as the so-called “ligandins”, a term that describes the non-substrate ligand-binding activity of GSTs towards endogenous molecules such as steroids, bilirubin, bile salts, and even toxic free heme [18,19]. By sequestering these metabolic by-products, GSTs reduce their intracellular bioavailability, thereby mitigating potential toxic side effects. This dual functionality—catalytic and reservoir/transporter—positions GSTs as central nodes in cellular detoxification networks and highlights their central role in maintaining cellular homeostasis.

For human GSTs, three families are described: cytosolic GSTs, mitochondrial GSTs, and membrane-associated proteins in eicosanoid and glutathione metabolism (MAPEG family) [16,17,18,19]. Cytosolic GSTs represent the most abundant and diversified family of this enzyme, being encoded by at least 16 genes categorized into seven distinct classes: alpha, mu, pi, sigma, theta, zeta, and omega, with genes from each class existing as dense clusters on specific chromosomes, most likely arising from ancient gene duplication events [20,21,22,23]. The first X-ray crystal structure of a human GST was reported for a pi class enzyme in 1992 [24], with subsequent crystal structures for all classes of human GSTs becoming available thereafter [19]. The comparative analysis of these structures revealed that, although amino acid sequences vary significantly—sharing only about 40% identity within the same class and as little as 25% identity across different classes—the protein structure, with the typical three-dimensional GST fold, remains remarkably conserved among all classes of cytosolic human GSTs [19]. They exhibit a highly conserved modular architecture, existing as dimers formed around a two-fold symmetry axis. Each monomer has on average a molecular mass of around 25 kDa, with 200 to 230 amino acid residues in length, and consists of two distinct domains: an N-terminal thioredoxin-like *αβ*-domain (Domain I or G-site) and a C-terminal all-*α*-helical domain (Domain II or H-site). The N-terminal domain is characterized by a four-stranded mixed *β*-sheet, while the C-terminal domain typically contains five to six major *α*-helices. However, the alpha, theta, and omega classes feature an additional *α*9-helix [19,25].

Dimerization is mediated by extensive interactions between the two subunits, often involving the so-called “lock-and-key” motif. This motif comprises an aromatic “key” residue from the loop in front of the *β*3 strand in Domain I of subunit 1 and a hydrophobic “lock” formed by the *α*4 and *α*5 helices in Domain II of subunit 2 [25,26]. In most *Hs*GST classes, this involves the amino acids Phe52 and Met51 being wedged into a hydrophobic pocket formed between the *α*4 and *α*5 helices, emerging from a loop between the *α*2 helix and the *β*3 strand [19]. While the absence of this wedge in the theta and omega classes highlights structural divergence, the dimeric state remains critical for maintaining protein stability and the catalytic integrity of the active site [19,25,27].

The catalytic activity of GSTs relies on two adjacent binding sites: the GSH-binding site (G-site) and the hydrophobic substrate-binding site (H-site). The G-site binds the tripeptide GSH in a conserved, extended conformation, anchored by hydrogen bonding with the *β*3*β*4*α*3 motif. Key interactions include the antiparallel alignment of GSH with the loop preceding strand *β*3 and the stabilization of the *γ*-glutamyl moiety by residues in the *β*4-*α*3 turn. Crucially, the sulfur atom of GSH is positioned at the N-terminal end of helix *α*1, where its hydrogen bonds with a catalytic residue—tyrosine, serine, or cysteine, depending on the enzyme class—to stabilize the activated thiolate (GS^−^) required for catalysis [25,28]. Conversely, the H-site is highly variable in topology and physicochemical properties, being formed by the loop between *β*1-*α*1, helix *α*4, and the C-terminal tail. This variability dictates substrate specificity, with the H-site characteristics ranging from the large hydrophobic cavities of the mu-class to the significantly smaller, hydrophilic pockets of the zeta-class [19,24,28].

### 3.2. Glutathione S-Transferases in Plasmodium Parasites

GST activity has been detected in the asexual blood stages (ABS) of several *Plasmodium* species, including the murine parasites *Plasmodium berghei* (*P. berghei*) and *Plasmodium yoelii* (*P. yoelii*), the zoonotic *Plasmodium knowlesi* (*P. knowlesi*) and *P. falciparum* [29,30,31,32,33,34]. These observations were further extended to and validated with recombinantly expressed GSTs from *P. berghei* [35], *P. falciparum* [32,35,36,37,38,39,40,41], and even *P. vivax* [39,42,43,44].

In *P. falciparum*, the characterization of the GST gene and respective protein was first achieved in the early 2000s [32,41]. This was identified as the product of a single-copy gene located on the left arm of the chromosome 14 sense (+) strand [45]. The gene was shown to contain a single intron and two exons, which are transcribed and further translated into a single cytosolic GST monomer of approximately 25 kDa that seems to act in a homodimeric fashion in vivo [32,41] and might represent between 1–10% of the total protein content in ABS [32]. Likewise, PvGST was shown to have the same dual-exonic gene architecture with a high degree of sequence homology, and an active state linked to a homodimeric quaternary structure [43,44]. To our knowledge, each species of *Plasmodium* contains a single GST isoform, a generalized claim made by visualizing gene synteny across the genomes of sequenced parasite species; however, most of the genes are putative and have no confirmed activity for a significant number of species and strains apart from those mentioned before [45]. By far, the most studied GST has been the one found in *P. falciparum* (PfGST, PF3D7_1419300), with which most of the structural and functional characterizations were achieved. This allowed us to show that this enzyme, in *Plasmodium* parasites, exhibits similarities but also important differences from its human counterparts that can be exploited for drug discovery.

Similar to other enzymes in the family, PfGST was earlier confirmed to promote the nucleophilic addition of GSH to known electrophilic substrates such as 1-chloro-2,4-dinitrobenzene (CDNB) and ethacrynic acid (ETA) [32,41]. Additionally, PfGST was also shown to exhibit low but important selenium-independent peroxidase activity against cumene hydroperoxide [32,41,46], suggesting its involvement in GSH-dependent peroxide detoxification, potentially compensating for the lack of a canonical GSH peroxidase in these parasites [11]. Yet, in the context of parasite physiology, the most important function might be the detoxification of free heme resulting from hemoglobin digestion. As shown by multiple works, in the presence of GSH, PfGST can be inhibited in a noncompetitive manner by hemin, indicating that this enzyme might be involved in hemin buffering in the infected erythrocyte [32,36,46]. Despite validated functional similarities, available data indicates a maximum of 37% sequence homology with known GST classes in diverse organisms [11,47] and only 24–34% sequence homology with human orthologs [32,37,48], which underlies particular structural and biochemical characteristics important for the discovery of antimalarials.

The first PfGST crystal structure was also resolved in the early 2000s, soon after the production of the first recombinant enzymes. The obtained crystal revealed unique structural aspects in PfGST that were different from most of the reported orthologs and therefore could not be assigned to any of the already known classes [37], despite three-dimensional structural analysis revealing an overall similar structural superposition with mu- and pi-classes of HsGST [46,49]. Early X-ray crystallography analysis has shown that although the PfGST monomer adopts the conserved GST fold and features a classical GSH binding pocket (G-site), the substrate binding site (H-site) is unusually solvent-exposed due to a shorter C-terminal sequence, suggesting that this structural adaptation in PfGST may account for a broader substrate specificity, in particular towards amphiphilic molecules [37]. Complementary three-dimensional analysis of PfGST structures, generated in the presence of the G-site-specific inhibitor S-hexylglutathione (GTX) [46,49], proposed that PfGST could be considered a “chimeric enzyme” since it gathers specific characteristics of different GST classes. PfGST was shown to have a rather rigid G-site, and its tertiary structure was not significantly affected by the bound inhibitor. However, this work validated the rather flexible C-terminus and showed that the absence of a classic mu-loop (typical of mu-class enzymes) should indeed be the reason why the H-site is more solvent-exposed and probably able to bind a wider range of substrates [49].

Another interesting structural feature of PfGST, which was also observed in PvGST and distinguishes them from other orthologs in the family (including HsGSTs), is the propensity to exist in solution as a tetramer rather than a dimer, which is significantly more stable, most likely due to its stronger ionic interactions. Nevertheless, the dimer is the active form of both PfGST and PvGST [44,46,48,50]. Importantly, these optimal physiologic functions seem to be primarily ensured by GSH concentrations, as this governs the tetramer-dimer equilibrium [44,46,50], probably by promoting rearrangements in the flexible loop (residues 113 to 119) located in the hydrophobic H-site, which prevents the dimers from locking. This particular event was suggested to be important for tetramerization and reduced enzymatic activity [36,51]. More specifically, the closely located Asn112 residue and Lys117 from complementary dimer subunits were hypothesized to engage in an interaction that requires Lys117 to undergo a complete positional rearrangement compared to its conformation in the dimer to ensure tetramerization, which was shown to be abrogated by a single mutation at the Asn112 residue (Asn112Leu) [51]. However, structural data generated by others challenged these assumptions by revealing the formation of tetramers in PfGST with a deleted 114–118 loop; yet, previous findings were not invalidated, as the authors postulated that loop rearrangement is a consequence but not the reason why tetramerization occurs [48]. Besides, GSH has also been shown to be important for the high-affinity cooperative binding of hemin to the active dimer but not essential for noncomparative binding, since the native enzyme, in the presence of oxidized GSSG, and the 113–119-loop mutants were able to bind hemin with lower affinity, indicating that the enzyme might be able to detoxify hemin in infected erythrocytes under significant oxidative stress [36]. The enzyme also exhibits pronounced positive homotropic behavior, wherein hemin binding to one subunit enhances the hemin-binding affinity of the adjacent subunit, thereby increasing the overall catalytic efficiency [40].

*Plasmodium* GST has proven importance for the detoxification of metabolic by-products such as hemin and might be the primary effector of this task, especially considering its abundance inside infected erythrocytes [32]. However, such activity is not exclusive to this enzyme; in *P. falciparum*, a protein known as exported protein 1 (PfEXP1, PF3D7_1121600) has also been shown to have GST activity and the capacity to rapidly and efficiently catalyze the conjugation of GSH to cytotoxic FP such as hematin [52]. Unlike PfGST, which has a cytosolic localization, PfEXP1 has been identified at the parasitophorous vacuolar membrane (PVM) [52,53]. PfEXP1 shares no significant sequence homology with cytosolic PfGST but exhibits functional convergence through independent evolutionary pathways [52]. By catalyzing its conjugation to GSH, PfEXP1 likely contributes to a membrane-associated detoxification pathway that prevents the harmful accumulation of non-crystallized hematin, serving a protective role distinct from hemozoin formation and apparently unique to this enzyme [54]. Further analysis of PfEXP1 substrate specificity has revealed striking differences in FP interaction compared to its cytosolic counterpart. While FP was shown to inhibit PfGST in a weak noncompetitive manner [46], hematin inhibition of PfEXP1 was shown to be competitive and twenty times stronger [52], suggesting that hematin may not merely inhibit PfEXP1 but could also function as a direct substrate, reinforcing its physiological relevance during the ABS. Despite being deemed dispensable in piggyBac insertion mutagenesis experiments [45,55], others have reported that EXP1 is indeed essential for the development of ABS in vitro. Homologous-recombination screens in *P. falciparum* failed to knock out the gene, indicating essentiality [56]. Functional importance was only addressed later on with conditional expression systems that showed impaired development of ABS when PfEXP1 expression was abrogated, which resulted from abnormalities and deficient nutrient transportation at the PVM [53,57]; complementation experiments with recombinant PfEXP1 bearing a mutation at the Arg70 residue, which impairs GST activity in this protein [52], rescued ABS development, suggesting that PfEXP1 GST activity, in particular, is not essential for parasite survival inside the infected erythrocyte [53,57]; this is different from *Plasmodium* GST, for which essentiality seems to be mostly linked specifically to GST activity (discussed later on in “Genetic validation” for Target Candidate Profiling).

Considering the aforementioned evidence, it can be concluded that, in the context of redox metabolism, some unique features of *Plasmodium* GST, but not EXP1, can be exploited for antimalarial therapies, which has been a topic of debate among the research community for several decades. Structural and functional uniqueness are characteristics that aid the development of candidate molecules with significant selectivity, mitigating potential side effects of the treatment. However, these aspects need to be evaluated in a broader context and under specific target candidate profiling criteria, considering, among several other points, the target essentiality in several stages of the *Plasmodium* life cycle.

## 4. Is *Plasmodium* GST Functionally Relevant Beyond the Blood Stages?

Most of the phenotypic characterization of GST has focused on *P. falciparum* ABS, which are easily propagated in vitro and for which scientists have developed a wide range of cellular and molecular tools to study them. However, this is not the case for other clinically relevant malaria parasites such as *P. vivax*, for which continuous in vitro culture remains unavailable despite ongoing efforts to overcome this limitation. Likewise, GST expression and function in other stages of the *P. falciparum* life cycle, such as the exoerythrocytic and sexual stages, have been poorly explored, mostly because studying these stages depends on expensive and often technologically advanced models. These include, for example, facilities for rearing and experimentally infecting mosquitoes, the use of non-human primate and humanized mouse models, and in vitro systems for culturing primary human hepatocytes [58,59,60].

Given these experimental constraints, indirect approaches are needed to assess whether GST functions extend beyond the ABS. One such approach is to look at transcriptomic data from orthologous genes in related *Plasmodium* species that are more amenable to study across the full life cycle when assays are available. The rationale behind this approach is based on comparative genomics; orthologous genes in different species descend from a common ancestral gene and tend to retain conserved functions, particularly when sequence similarity is high [61]. Therefore, evidence of GST ortholog expression in non-ABS of other *Plasmodium* species may provide useful indirect evidence of GST activity throughout the life cycle.

**Figure 1:**
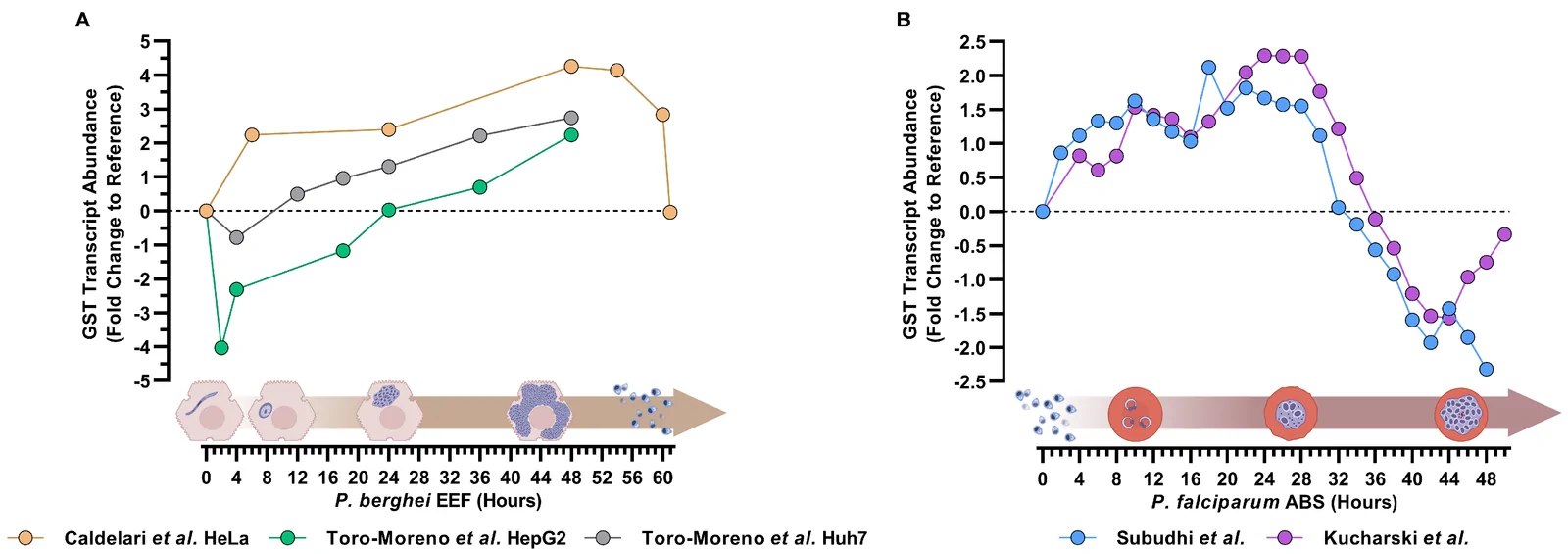
An overview of GST expression trends across the major replicative stages of *Plasmodium* life cycle. Transcripts per million (TPM) of (**A**) PBANKA_1023900 in the exoerythrocytic forms (EEF) of *P. berghei* [62,63] and (**B**) PF3D7_1419300 in the asexual blood stages (ABS) of *P. falciparum* [64,65] were normalized to TPM of the reference invasive forms (**A**) sporozoites and (**B**) merozoites (timepoints 0 and 52 h in Subudhi et al. and Kucharski et al., respectively) collected within the same study. TPM ratios were base-2 log transformed plus 0.001 and plotted as a function of time of their respective stage development, where each unit variation in the Y-axis represents a duplication in GST transcript abundance relative to transcript levels of the respective invasive form. Averaged TPM values are available and were repurposed from the PlasmoDB transcriptomics tool [45] for each of the aforementioned reference genes. (**A**) *P. berghei* EEF: Sporozoites (0 h), trophozoites (1–24 h), schizonts (24–48 h) and merozoites (48–60 h). (**B**) *P. falciparum* ABS: Merozoites (0 h), rings (1–16 h), trophozoites (16–36 h) and schizonts (36–48 h). Created in BioRender. Wrenger, C. (2026) https://BioRender.com/fsrr46z (accessed on 29 June 2026) and GraphPad Prism version 8.4.3 for Windows (GraphPad Software, San Diego, CA, USA, www.graphpad.com).

As such, the following discussion is mostly based on searching and interpreting transcriptional data of PfGST and its relevant orthologs as curated by PlasmoDB, an open-access bioinformatics tool that compiles, trims, aligns and curates transcriptomic data from published databases for intuitive visualization and research [45]. GST transcript abundance in the *P. berghei* liver stage (LS) (reference PBANKA_1023900, Figure 1A) and *P. falciparum* ABS (reference PF3D7_1419300, Figure 1B) was analyzed to aid discussion. Originally published research that generated the databases was consulted for experimental interpretation and discussion. However, most of the data discussed here derives from bulk RNA sequencing experiments with limited sample sizes. Consequently, our conclusions are drawn with appropriate caution and will require further validation through complementary experimental approaches.

### 4.1. Asexual Blood Stages

As previously mentioned, the possibility of culturing *P. falciparum* ABS has led to generating a disproportionate amount of data compared to other stages of the life cycle. Consequently, most of the aforementioned phenotypic characterization of PfGST has been limited to ABS. Likewise, several RNA sequencing databases were generated for several timepoints of the intraerythrocytic development. Establishing this baseline expression pattern is important, as it provides a reference point for evaluating potential GST function in other stages of the life cycle.

Multiple studies that generated bulk RNA sequencing data from tightly synchronized *P. falciparum* in vitro cultures, regardless of the sequencing technology in place, have shown that PfGST transcript abundance (reference PF3D7_1419300) peaks within the 16–36 h timepoints of the intraerythrocytic development (Figure 1B), which coincides with the trophozoite stage in the blood [64,65,66,67,68,69,70,71,72]. In general, transcript abundance seems to increase progressively after merozoite invasion and ring formation, reaching its maximum expression during the trophozoite stage and then steadily declining during schizogony until the formation of merozoites. Variation in the expression patterns observed across studies seems to be negligible, with the major expression trends not being affected by experimental design or conditions that are known to affect the expression patterns of other types of genes in *P. falciparum* ABS (e.g., the clonally variant *stevor* gene family) [71]. These trends were also observed for the orthologs in *P. vivax* ABS cultured ex vivo (reference PVP01_1329600 [P01 strain] or PVX_085515 [Sal-1 strain]) [73] and *P. berghei* isolated from infected mice (reference PBANKA_1023900) [74].

Differential expression of GST during the ABS might indicate higher GST dependency and, consequently, essentiality for that stage given the aforementioned reported functions. In fact, this was demonstrated in pharmacodynamic studies, which showed a predominant inhibition of trophozoites, among all the ABS, by the broad GST inhibitor ellagic acid (ELA) and a chemically modified analog [75,76], confirming that GST is mostly essential for blood stage trophozoites, despite early counterintuitive evidence suggesting that, among the three main ABS, trophozoites would be the ones with the least GST activity [33].

### 4.2. Exoerythrocytic Stages

The presence of PfGST transcripts (reference PF3D7_1419300) has been detected in sporozoites of *P. falciparum* [77,78], as well as its ortholog PvGST in sporozoites of *P. vivax* (reference PVP01_1329600) [79,80]. The abundance of these transcripts has been detected at various levels depending on the study design and RNA sequencing technology in place; yet some comparative studies have been able to capture some differential expression patterns between stages. In *P. falciparum* sporozoites, PfGST transcript levels (reference PF3D7_1419300) appear to remain stable as sporozoites travel from midgut oocysts to salivary glands (10 vs. 14 days post-infection) [77]; however, transcript data obtained in other studies suggest that PfGST expression in oocysts might be higher than in salivary gland sporozoites [78,81]. These discrepancies might be explained by the timing of sampling: assessing PfGST expression at early oocyst development timepoints (7–8 days post-infection) might have coincided with ongoing sporogony. Importantly, looking at data generated within the same transcriptomic study, PfGST expression in sporozoites is lower than in ring stage parasites [78]. Others have found contradictory evidence, suggesting that the *P. vivax* GST (reference PVP01_1329600) was upregulated in sporozoites when compared to blood stages [80]; however, these findings relied on comparisons done between transcript levels quantified for the different stages across different studies, which the authors validated by the observation that some “housekeeping” genes presented a reduced expression variability across stages and this was reproducible across the studies; the authors concluded that any hypothetical variability associated with the “batch effect” would be sufficiently lower than the real gene expression differences observed between stages [73,80]. Nevertheless, this assumption might not hold true for enzymes such as GST that (1) are not constitutively expressed at the same intensity across different timepoints within the same stage (e.g., throughout liver and blood stages), and (2) since sporozoite transcript levels in that particular study were compared to mixed blood stage cultures.

The apparent lower expression of GST in mature sporozoites might indicate non-essentiality for this stage due to the non-replicative and more invasive nature of this parasite form; yet this could not be addressed in homologous-recombination screens done with *P. berghei* mutants due to possible heterozygous rescue in the mosquitoes upon cross-fertilization events between uptaken clones [45,82]. This idea is endorsed by the observations of increasing GST expression after sporozoite invasion of host cells and throughout LS development. Transcriptional levels of the PfGST ortholog in *P. berghei* (reference PBANKA_1023900) seem to be higher in post-invasion parasites than in pre-invasion sporozoites. Transcript abundance seems to reach its highest around the 48-h timepoint post-invasion of HeLa (cervical cancer-derived cells) [63], HepG2 and HuH7 cells (hepatoma-derived cells) [62,83], which coincides with the trophozoite to schizont transition in intrahepatic *P. berghei* parasites (Figure 1A). Transcription of this ortholog gene seems to decrease after schizont development and towards parasite egress from host cells. Data obtained from *P. berghei*-infected HeLa cells suggests that merosomes-containing merozoites have comparable PBANKA_1023900 transcript levels to pre-invasion sporozoites [63], further supporting the hypothesis that GST function might be restricted to replicative rather than invasive forms of the parasite.

Transcripts of PfGST orthologs were also found in non-replicative hypnozoites of *P. vivax* (reference PVP01_1329600) [84] and *Plasmodium cynomolgi* (*P. cynomolgi*) (reference PCYM_1334900) [85], which are quiescent forms of these parasites that cause relapsing malaria in humans and primates, respectively. In the first study, no clear differences were observed in PVP01_1329600 transcript levels between mixed *P. vivax* LS cultures and those enriched for hypnozoites using phosphatidylinositol-4-OH kinase (PI4K) inhibitors, which promote the selective depletion of developing schizonts [84]. However, advanced stage-separation techniques in the *P. cynomolgi* model have yielded clearer insights into the putative GST expression across these developmental phases. Fluorescence-activated cell sorting (FACS) of GFP-expressing *P. cynomolgi* parasites allowed the purification of hypnozoites and developing schizonts based on their differential GFP expression at 6 days post-infection of primate hepatocytes. RNA sequencing of these forms has shown a statistically significant upregulation of the PfGST ortholog PCYM_1334900 in developing schizonts when compared to hypnozoites [85]. Likewise, RNA sequencing data generated for *P. cynomolgi* hypnozoites and schizonts isolated from the livers of infected *Macaca fascicularis* using laser capture microdissections also helped to overcome transcript contaminations intrinsic to samples containing mixed liver stages [86]. This study detected transcripts of that PfGST ortholog in both *P. cynomolgi* liver schizonts and blood stages (original manuscript alignment–reference PCYB_133790; PlasmoDB alignment–reference PCYM_1334900) [86], which were undetected in hypnozoites. In line with these observations, the recently synthesized CB series of compounds, designed to specifically target the GST H-site, failed to convincingly kill hypnozoites in an in vitro LS assay with *P. cynomolgi* and primate hepatocytes, despite their significant potency against *P. falciparum* ABS and milder activity against other stages of the *Plasmodium* life cycle [35].

Taken together, these findings reinforce the hypothesis that the expression of GST is reduced in non-replicative forms of *Plasmodium* parasites and upregulated during asexual replication, where it is essential for redox homeostasis.

### 4.3. Sexual Stages

PfGST transcripts (reference PF3D7_1419300) were also detected in *P. falciparum* blood gametocytes of both sexes [87] and for multiple stages (stage II and V) [88]. In the latter, PfGST transcripts were not highlighted as regulated in the original manuscript [88] but were identified at low levels in the PlasmoDB analysis, which might be a consequence of reduced sequencing depth since trophozoites are also shown to have reduced transcript counts per million (TPM) in that study. The regulatory orientation of these transcripts in gametocytes in relation to other ABS is inconclusive since results varied with the methodology used for library preparation (strand-specific unidirectional vs. non-stranded bidirectional) [88]. Nevertheless, the gene seems to be transcribed in gametocytes without being significantly differentially regulated between males and females. The corresponding protein was detected by liquid chromatography tandem mass spectrometry (LC-MS/MS) in both sexes, suggesting a lack of regulation through translational repression at this stage [87,89]. Other studies confirmed previous observations done in *P. falciparum* by detecting transcripts of the PfGST ortholog (reference PBANKA_1023900) in *P. berghei* gametocytes of both sexes [74,90]. As before, methodology affects inferences regarding differential gene expression between gametocytes and other ABS since, for those reporting such approaches, ABS preparations that were sequenced contained mixed asynchronous populations, which may skew data interpretation [90]. Despite reduced metabolic activity and observations of ceasing hemoglobin digestion towards the mature stage V of gametocytogenesis [91], the apparent moderate expression of GST observed here might be responding to basal or non-metabolic sources of oxidative stress. In fact, mature gametocytes were shown to be susceptible to the inhibition of glutathione reductase (GR) activity, a key enzyme that feeds the GSH pool in an NADPH-dependent manner, suggesting that glutathione metabolism, and possibly GST, might be important for stage V gametocyte responses to oxidative stress [92]. We believe that the unsuccessful inhibition of gametocyte activation reported for the compounds in the CB series mentioned before [35] does not invalidate GST importance for late-stage gametocytes since additional tests are required for that purpose (discussed later on in “Assay readiness” for Target Candidate Profiling).

Interestingly, the presence of GST transcripts was scarce or even absent in both *P. falciparum* and *P. berghei* ookinetes [74,88], the parasite form resulting from gamete fertilization and zygote development in the mosquito that invades the insect midgut wall to form oocysts and initiate sporogony. These observations support our initial hypothesis that the expression of GST might be restricted to replicative stages; however, compounds targeting the GST H-site were suggested to kill ookinetes in a dose-dependent manner [35]. As such, it is possible that *Plasmodium* GST is functionally important for non-replicative gametocytes and ookinetes in a way that cannot be predicted from evaluating transcript abundance.

## 5. Revisiting *Plasmodium* GST as a Potential Target for New Antimalarials: Opportunities and Limitations

### 5.1. Target Candidate (TCP) and Product (TPP) Profiling

As mentioned earlier, treating patients infected with *Plasmodium* to eradicate malaria is a complex task that requires a multidisciplinary approach to overcome the different epidemiological challenges (e.g., resistance to antimalarials, cryptic parasite forms, etc.). This crisis emphasizes the need to understand these mechanisms and develop appropriate counter-strategies. Therefore, to increase the effectiveness of newly developed drugs, it is important that the unique biological characteristics of each stage of the *Plasmodium* life cycle are considered as these may offer the most effective targets for new therapeutic interventions with different modes of action [5,10].

As Forte and coworkers from MalDA (Malaria Drug Accelerator) analyzed, the main focus in the search for new molecular targets for antimalarial drug discovery needs to be multistage targeting according to the Target Product Profile (TPP) definitions [10]. In 2013, TPPs were first suggested for malaria as the optimal and minimally acceptable characteristics of a combination medicine required to kill a complex multistage organism such as *Plasmodium*; by definition, this medicine should result from a combination of candidate molecules with ideal and minimally acceptable features for multistage targeting defined in the Target Candidate Profile (TCP) [93]. Ideally, a good target-compound pair would check a combination of features described by the TCP definitions and be validated for either 1) treating patients suffering from the active disease in combination with a strategy against relapses (ABS and LS hypnozoites) and/or transmission blocking (ABS and gametocytes) (TPP-1 or 2) chemoprotection and prophylaxis focusing on treating and preventing malaria in vulnerable populations and outbreaks (ABS and LS) (TPP-2) [10] (Table 1A). After evaluating publicly available transcriptomic data to extrapolate GST functions beyond the ABS, we hypothesize that the available GST-compound pairs and those that are yet to be developed could meet both TPP-1 and TPP-2 criteria and be used in the treatment of active disease and for chemoprotection, respectively (Table 1B).

GST-compound pairs could intuitively meet the criteria defined in TCP-1 by targeting ABS, supported by its high transcript abundance in blood stage trophozoites [64,65,66,67,68,69,70,71,72], which seems to be essential for their development, as proven by piggyBac insertion mutagenesis experiments [45,55] and in vitro proliferation experiments using ABS with available compounds targeting GST [35,76,94]. The low to null expression of PfGST orthologs in *P. vivax* and *P. cynomolgi* hypnozoites [84,85,86] supports the idea that this target would not be suitable for developing anti-relapse compounds that could be used for the radical cure of *P. vivax* malaria. Moreover, hypnozoites are absent in the *P. falciparum* life cycle, further invalidating TCP-3 for the discovery of compounds against this species. Nevertheless, despite the lack of proven expression in developing *P. falciparum* LS, transcripts of putative PfGST orthologs have been detected in *P. berghei*, *P. cynomolgi* and *P. vivax* exoerythrocytic trophozoites and schizonts [62,63,83,84,85,86], suggesting a role for GST in LS development and an opportunity to meet the TCP-4 requirements for prophylaxis.

Likewise, PfGST transcript and protein were detected in gametocytes, especially in the mature stage V [87,88,89], which might be important since molecules targeting other steps of GSH metabolism have been shown to inhibit the development of these later stages [92], suggesting an opportunity to meet TCP-5. On the other hand, since this is a parasite-specific target without a significant number of small molecules available, it is difficult to foresee how target–compound pairs that are yet to be developed could meet TCP-6 by jointly affecting the vector (e.g., mosquitocides and endectocides). Additionally, since GST seems to have reduced to null expression in invasive ookinetes and sporozoites, we believe that it might be difficult for GST-compound pairs to meet TCP-6 requirements. However, recent evidence suggests that ookinetes can be killed with GST-specific compounds [35].

Altogether, after target assessment for prioritization, we believe that GST might be a good alternative target to explore the development of new antimalarials while meeting the requirements for treating active disease (TPP-1) and chemoprevention (TPP-2) [10].

### 5.2. Target Assessment: Can GST Meet the Criteria for Target Prioritization?

So far, we have suggested PfGST as a good target candidate for new antimalarials by validating its suitability for several TCPs and TPPs based on its expression and essentiality to one stage of the *P. falciparum* life cycle. However, prioritizing a target requires an exhaustive assessment of several criteria that facilitate its ranking, as previously suggested by the MalDA consortium [10], which we summarized in Table 2.

#### 5.2.1. Genetic Validation

Tier 1 requirements for target prioritization start with genetic validation experiments, ranking its priority as medium or high depending on the available evidence. Accordingly, GST was validated as essential for *P. falciparum* ABS development in vitro as shown in genome-wide saturation mutagenesis experiments, with the GST mutant presenting a mutagenesis index (MIS) and fitness (MFS) scores of 0.12 and −3.2, respectively [45,55]. On the other hand, the GST ortholog in *P. berghei* was deemed dispensable for in vivo ABS and LS development of this species, as shown in homologous-recombinant knockout experiments; essentiality for the mosquito-to-salivary gland transition was inconclusive due to a lack of power occurring as a consequence of possible heterozygous rescue upon fertilization [45,82,96]. However, some of these findings in ABS were later disproved with the confirmed existence of a druggable PfGST ortholog in the murine parasite; the protein was deemed essential for *P. berghei* ABS since multiple attempts to knockout the *pbgst* locus were refractory, confirming essentiality [94]. Additionally, mutants generated by large-scale homologous recombination screens were not genotyped [82,96], a concern raised by others as they might have failed to integrate into the *pbgst* locus of interest [94]. With the available evidence, GST can be considered a valid target with medium priority score for genetics since it requires additional experimental validation (e.g., conditional knockouts/knockdown).

#### 5.2.2. Chemical Validation

When targets are unavailable for biochemical assays and for the development of cocrystal structures, which is often the case with *Plasmodium*-related proteins due to the difficulties underlying recombinant protein production in this organism, in vitro resistance induction with sublethal doses of active molecules is often used to induce resistance, followed by whole genome sequencing (WGS) to identify the mutated locus in the new resistant strain and consequently validate the target [10]. However, this was not accomplished with the available molecules targeting *Plasmodium* GST (discussed in Section 5.2.3). Consequently, no transgenic parasites bearing hypothetical resistance-related mutations are available, in which susceptibility to available molecules should vary if the modified locus encodes the target with fitness-improving mutations [10]. In contrast, since recombinant versions of *Plasmodium* GST are widely available, molecules against this enzyme have been largely validated with biochemical assays, followed by attempts to establish good correlations between the inhibitory activity of the enzyme and parasite development in vitro. Molecules developed specifically against the H-site of *Plasmodium* GST have shown encouraging correlations between enzymatic and phenotypic in vitro assays, while providing dose-dependent inhibition with important levels of selectivity (discussed in more detail in Section 5.2.4). For instance, the H-site specific CB-27 molecule inhibited *Pb*GST activity in a dose-dependent manner, with a non-disclosed IC50 estimated at around 10 μM, which correlated with activity against *P. berghei* and *P. falciparum* Dd2 ABS, with EC50s predicted at around 505 and 958 nM, respectively [94]. Later on, CB-27 inhibition of PbGST and PfGST was tested in dose–response assays, and IC50s were estimated at 21.1 and 22.9 μM, respectively, which correlated nicely with activity against several *P. falciparum* strains, with EC50s spanning between 280 and 400 nM [35]. However, compounds in the series derived from CB-27 did not show the same correlation between enzyme and in vitro ABS activity, although some compounds in the series, such as CB-61, were shown to be considerably potent against several *P. falciparum* strains, with reported EC50s spanning between 200 and 300 nM [35]. Another small series of two analogs, flavellagic acid (FEA) and coruleoellagic acid (CEA), derived from the G-site specific inhibitor ellagic acid (ELA), which were chemically engineered towards the H-site, have also shown improved correlations between enzyme and *P. falciparum* ABS activity; FEA and CEA have reported PfGST IC50s of 29.3 and 7.0 μM, respectively, while showing strain-transcendent effects spanning between 94 and 186 nM for FEA and 42 to 116 nM for CEA. Importantly, for this particular study, differences in potency between CEA and FEA against PfGST were preserved when transitioning to in vitro ABS assays, with CEA always being the most potent compound regardless of the assay, suggesting targeting coherence [76]. Unfortunately, some of these series have a small variety of compounds, or not all are effective in both biochemical and phenotypic in vitro assays, which impairs the establishment of correlations between effects against recombinant enzymes and in vitro growth inhibition over 3 log units for a compound series as suggested by the MalDA [10].

From our perspective, significant improvements need to be made to enhance the chemical validation of this target, which is mostly intertwined with the synthesis and availability of compound series with a wider range of molecular entities that explore novel areas of the related chemical space. Nevertheless, existing results might encourage future development in this area, as we believe that *Plasmodium* GST can still have a medium score for chemical validation due to the existing number of compounds known for targeting the recombinant enzymes.

#### 5.2.3. Resistance Potential

Assessing the risk of developing resistance in a given target–compound pair is of extreme importance, considering that one of the main challenges for eradication is the worldwide distribution of resistance to available antimalarials, especially in ABS [4,5]. To assess this, in vitro resistance induction experiments with ABS, pressured by sublethal concentrations of a given molecule, are required to estimate the minimum inoculum for resistance (MIR), which is often used as a proxy for resistance potential. MIR >8 is preferable, indicating that at least 10^8^ parasites are needed to develop resistance. If this threshold is not achieved, the target–compound pair can be deemed “irresistible” or otherwise result in resistant strains with increased EC50 against the compound [10,106]. Unfortunately, no such experiments have been reported, but these are necessary to evaluate the resistance potential of available molecules (i.e., CB-27, CB-61, FEA and CEA [35,76,94]).

Resistance can arise as a result of several events, including gene amplification, de novo mutations and selection of preexisting single nucleotide polymorphisms (SNPs) present in a given parasite population, which also justifies the need for target-compound validation using clinical isolates in the field [106]. There are no mutations in *Plasmodium* GST known to improve fitness against available compounds, yet assessing the abundance of SNPs in the gene may provide indirect evidence for the potential of selecting resistance in this target. When evaluating *pfgst* gene sequences in more than 200 genomes of geographically spread *P. falciparum* isolates, available in the PlasmoDB database [45], a total of nine SNPs could be identified in the coding region of the gene. While three of these SNPs are of synonymous nature (sSNPs), six non-synonymous SNPs (nsSNPs) are reported, which result in a single amino acid substitution in the protein sequence with varied prevalences (Val6Ala [12%], Leu67Met [1%], Phe135Leu [3%], Thr174Ala [0.4%], Glu191Asp [1%] and Val210Ala [0.4%]). Importantly, the listed substitutions do not overlap with residues reported to be relevant for PfGST catalytic activity, such as Tyr9, Lys49, Val59, Gln71, Ser72 and Asp105 (G-site), as well as Phe10, Phe42 and Phe45 (H-site) [37,46,48]. In line with this observation, others reported an exact protein sequence among PvGST from 15 geographically different *P. vivax* field isolates [43]. This, along with the low worldwide prevalence of nsSNPs found outside relevant PfGST residues, might indicate that the catalytic sites are naturally under reduced selective pressure, suggesting a lower risk for developing/selecting resistance against molecules targeting the enzyme.

Additionally, target–compound pairs must be validated against the ABS of sensitive and multidrug-resistant strains of *P. falciparum* to assess the hypothetical risk of cross-resistance mediated by resistance mechanisms associated with known antimalarial targets (i.e., *pfcytb* and *pfdhfr*) or transporters (i.e., *pfcrt* and *pfmdr1*) [106]. Important compounds in the CB series such as CB-27 and CB-61 were shown to inhibit the ABS of *Pf*Dd2 (*pfcrt*, *pfmdr1* and *pfdhfr*) and *Pf*C2B (*pfcytb*), while CEA and FEA were also shown to inhibit the ABS of *Pf*Dd2 and *Pf*K1 (*pfcrt*, *pfmdr1* and *pfdhfr*) [35,94], which are part of a geographically diversified multidrug-resistant panel of strains suggested to be included in cross-resistance studies [106]. Some of these compounds were also successfully tested against other relevant strains, including *Pf*W2, *Pf*TM91c235, *Pf*HB3, *Pf*F32, *Pf*FcB1 and *Pf*FcM29 [35,75,76], yet there are no reports of ABS inhibition of artemisinin-resistant strains bearing *pfkelch13* mutations.

Lastly, some additional important features of this enzyme that are worth mentioning and add to the prioritization checklist in this section are (1) the significant degree of conservation and sequence homology among *Plasmodium* species, especially in the H-site region, which is important for the transspecies efficacy of a compound, and (2) being encoded by a single-copy gene, which in turn gets translated into a unique multifunctional enzyme, whose inhibition would unlikely be rescued by compensatory mechanisms [43,45].

Overall, despite the missing empirical characterization of mutations conferring resistance in *Plasmodium* GST, evidence indicates a low risk for resistance upon drug targeting, which is aligned with its essential role in the parasite, where mutations may impose significant fitness costs. Therefore, *Plasmodium* GST should progress to tier 2 assessment despite scoring low in this criterion for target prioritization.

#### 5.2.4. Druggability

GST is a valid target according to the tier 1 requirement list. As such, it can progress to tier 2 assessment, which initiates with target druggability validation. Within this category, targets for which small molecule inhibitors have been identified receive the highest priority score [10], which is the case for *Plasmodium* GST. Virtual library screens using the *P. berghei* GST 3D structural homology model allowed the identification of 40 small molecules that could “dock” to either one of the two catalytic sites. CB-6 and CB-19 (G-site) inhibited *P. berghei* ABS with an EC50 >20 μM, while CB-27 (H-site) inhibited both *P. berghei* and *P. falciparum* ABS with an EC50 <1 μM. CB-27 was then used as a scaffold to identify new molecules in silico by Rapid Overlay of Chemical Structures (ROCS), creating the hydrazone CB series of compounds. This series was predicted to have a positive absorption, distribution, metabolism, and excretion (ADME) profile; however, compounds in the series were predicted to have at least one of the undesirable toxicity effects (T) tested: hepato- and cardiotoxicity, and carcinogenic potential. No hemolytic activity was observed, further validating the specific ABS effect [94]. The activity of the CB series was tested in future work against different parasite species and stages of the life cycle to validate TCP/TPP fit, making them the first compound series in the GST drug development pipeline. Nevertheless, MedChem is still required to optimize their characteristics if these are to be announced as potential candidates [35].

Despite these contemporary findings, researchers have been looking for unique molecules that could selectively inhibit *Plasmodium* GST for more than two decades. Most of these studies report promising attempts and even describe “hits” that may have the potential to be developed. Despite using either rational drug design or traditional chemical synthesis approaches, most of these studies rely solely on assays against recombinant enzymes or crude parasite extracts, whose effects have poorly correlated with in vitro efficacy tests with live parasites. One of these early studies, in particular, reports the generation of a significant library of synthetic compounds from which a few inhibit GST activity in either *P. yoelii* crude extracts or recombinant PfGST, while having an apparent significant efficacy against *P. falciparum* ABS. Some compounds in the synthesized series (e.g., No. 14, 80, 82 and 83) are described as having 100% in vitro efficacy against *P. falciparum* at 100 nM; yet, proper proliferation assays and detailed methodological descriptions are missing, impairing the accurate assessment of compounds’ efficacy and potency and eventually their suitability for entering the drug development pipeline [29].

A significant part of the studies has relied on exploring natural products as a source of unique scaffolds that could enhance the synthesis of promising analogs. A range of products isolated from medicinal plants, such as JBC 42C (sesquiterpene), Tral-1 (bicoumarin), ELA and curcumin, have been shown to inhibit recombinant PfGST in the lower micromolar range [38]. However, additional experiments with recombinant HsGST, competition binding assays, or in silico computational analysis were missing to infer selectivity or the binding sites driving inhibition. ELA, for instance, a natural compound found in edible plants with known anti-carcinogenic, anti-oxidant and anti-inflammatory properties, has been extensively studied as a *Plasmodium* GST inhibitor [38,75,76,94]. However, competitive assays with GSH and CDNB indicated that PfGST inhibition by ELA occurs at both the G- and H-site [38], an apparent characteristic of broader GST inhibitors and a potential issue for selectivity. In fact, ELA was shown to equally inhibit recombinant Pf and HsGST, disqualifying it as an antimalarial [76], despite reports suggesting low levels of toxicity against mammalian cells in vitro [75,103,104]. Yet, the ELA analogs FEA and CEA presented improved inhibition of the recombinant parasite enzyme (<30 µM) and *P. falciparum* proliferation in vitro (<200 nM for several strains), with additional improvements in selectivity and reduced toxicity in mammalian cells by several orders of magnitude, which was achieved by chemically directing the molecule towards the solvent-accessible and less conserved H-site [76]. Additionally, ELA and CEA were shown to reduce parasitemia in mice infected with murine parasites, marking the first demonstration of in vivo antimalarial activity for compounds targeting GST [75,76]. These works suggest that existing compounds, and more importantly, natural products, can provide useful scaffolds that can be improved by MedChem to generate new molecules against *Plasmodium* GST.

Likely inspired by phytochemicals, alternative approaches have predicted small molecules targeting GST with encouraging ADMET scores in silico, but the lack of experimental evidence regarding their efficacy diminishes their score in the priority list. Molecular docking studies with phytocompounds from *Azadirachta indica* (“Neem”), a tropical evergreen tree native to the Indian subcontinent and some parts of Southeast Asia, have predicted promising *Pf*GST inhibitors in the IMPHY library, including IMPHY000093, IMPHY001448, and IMPHY005310, among others, which had a high predicted binding affinity to the PfGST G-site (low docking scores) [98]. Although encouraging, the compounds may not pass selectivity tests since binding was predicted to occur at the less diversified G-site. Similarly, *Plasmodium* GST was predicted to be one of the most favorable targets of knipholone, a phytocompound isolated from the roots of the Ethiopian medicinal plant *Kniphofia foliosa*, which has been reported to have antiplasmodial activity [97]. Also, baicalin (BA) and 5,7,3′-trihydroxy-6,4′,5′-trimethoxyflavone (TTMF) were initially predicted, among the flavonoids in the MedChemExpress database, to be some of the best ligands for PfGST and PvGST, respectively [39]. The inhibition potential of these molecules was empirically validated using recombinant Pf/PvGST in enzymatic activity assays. Complete inhibition of both enzymes was achieved with BA and TTMF, with minimal effect on the three recombinant HsGST isoforms tested. These molecules, which were predicted as H-site-specific binders, inhibited recombinant *Plasmodium* GST with IC50 values in the low micromolar range and no predicted off-target effects, highlighting them as possible candidates for further research and development (e.g., in vitro and in vivo efficacy and ADMET studies) [39]. BA and TTMF outperformed bromosulfophthalein (BSP) in enzymatic activity assays with recombinant Pf/PvGST. BSP is an anionic phthalein triphenylmethane dye used to study liver injury and transmembrane transport of GSH, but it is also a proven inhibitor of Pf/PvGST, apparently through binding to the H-site. However, this molecule is unlikely to be prioritized for development due to the lack of empirical validation, poor solubility in water and predicted difficulties for biliary excretion due to its considerable molecular weight (>500 g/mol) [42].

Other broadly used molecules in the field of GST research, such as GTX [32,38,94], ETA [32,38], cibacron blue (CB) [30,32] and even chloroquine (CQ) [32,33,46], are also known inhibitors of *Plasmodium* GST but are unlikely to enter a development phase for the purposes discussed here. CQ inhibition of GST activity is controversial, since it was demonstrated in extracts of synchronized *P. knowlesi* ABS [33] but later undermined by observations made in enzymatic assays with recombinant PfGST [32] and *P. falciparum* trophozoite extracts [46]. Nevertheless, CQ targeting of GST for clinical use is invalidated due to the worldwide distribution of *pfcrt* mutations, which confer CQ resistance to *P. falciparum* strains [4,5]. Likewise, some of the aforementioned compounds, such as GTX, ETA and CB, are promiscuous GST inhibitors, binding to both *Plasmodium* and human enzymes [32]. In silico predictions suggest the existence of competitive binding with the CDNB substrate, but also with GSH, indicating binding to both H- and G-sites, respectively, which might hinder compound selectivity [100]. In fact, some of these molecules have been suggested for anticancer therapies [17,107], highlighting their potential off-target effects in humans if used to treat malaria. However, it might be possible to chemically modify them to improve selectivity, as previously done with ELA [76]. Otherwise, the application of these molecules might include their usage as “tool compounds” in the validation of *Plasmodium* GST as a target for future identified molecules.

The synthesis of peptide inhibitors has also been explored as an alternative strategy to target *Plasmodium* GST. In the early 2000s, the first PfGST crystal structure revealed the uniqueness of the H-site pocket, including the identification of a peptide loop connecting both GSTs in the homodimeric structure. This inspired the first reported de novo synthesis of inhibitory peptides with encouraging levels of selectivity against the enzyme [37]. More recently, and despite potential selectivity issues, de novo design of fragments was done with the G-site as a target, as well as GSH and GTX as scaffolds; the objective was to disrupt heme binding to PfGST under the assumption that free heme would amplify oxidative damage to the parasite. This work proposed a wide selection of in silico fragments with acceptable affinity and physicochemical properties; however, these compounds were not synthesized, and empirical validation with cocrystals or in vitro assays is missing [99].

Overall, available crystal structures of *Plasmodium* GST have led to a significant amount of work underlying computational analysis of potential “hits” that, most of the time, were not empirically validated; in theory, target–compound pairs would score lower on the priority list. However, other GST-specific molecules with drug-like properties (e.g., CB series, ELA analogs, etc.) have been tested in biochemical and phenotypic in vitro screening assays, as well as in efficacy tests in vivo, suggesting that the target can be drugged in an encouraging number of systems, increasing its score on the priority list and setting the foundation for testing against different stages of the life cycle and empirically evaluating the TCP/TPP fitting.

#### 5.2.5. TCP/TPP Fit

We have previously discussed the hypothetical importance of *Plasmodium* GST in and beyond the ABS; publicly available data pinpoints the transcription of GST in ABS, developing LS, and maybe late-stage gametocytes, suggesting an opportunity to meet TPP-1 (TCP-1 & TCP-5) and TPP-2 (TCP-1 & TCP-4). However, fewer studies have actually validated the TCP/TPP fitting in efficacy tests with the already available compounds.

At this point, it is clear, and there is a consensus among the research community, that GST targeting during the ABS is possible. As a matter of fact, several authors have reported in vitro efficacy of various compounds against *P. falciparum* ABS using standard proliferation assays [35,75,76,94]; these were only done using conventional 72-h assay formats, and no additional testing was performed to validate these compounds as fast-acting molecules. However, some of those compounds have proven activity against several *P. falciparum* strains bearing resistance to a wide range of antimalarials [35,75,76,94], which is an important consideration for TCP-1 validation [10]. Additionally, Peter’s 4-day suppressive test in a rodent model infected with *Plasmodium vinckei* indicates that an unoptimized compound such as ELA, administered intraperitoneally, can suppress peripheral parasitemia in vivo without a risk of recrudescence, further validating GST as a promising target in ABS [75].

Empirical evidence for the usage of GST-compound pairs in other TCPs is only available in a recent work, which reported the activity of the CB series against *Plasmodium* across several stages of its life cycle [35]. As a starting point for this discussion, it is important to consider that most of the compounds in the series were active against *P. falciparum* ABS, especially CB-27 and CB-61, which have reported EC50s as low as 200 nM, depending on the strain. In contrast, none of these compounds were active against *P. cynomolgi* LS, with the exception of CB-41, which had a reported EC50 of approximately 8.6 μM in a prophylactic treatment scheme (a 4-day treatment after sporozoite inoculation) but showed no effect in a radical cure regimen (when liver schizonts and hypnozoites are formed). Differences in the *P. cynomolgi* ortholog might explain the varying effects of the compounds in the LS; however, technical replicates are often difficult to execute in this model, which, together with a reduced number of independent observations and the lack of an effect in the radical cure mode, might lead to an overestimation of the CB-41 effect on *P. cynomolgi* LS [35]. In vitro models using HepG2 cells infected with *P. berghei* luminescent parasites might represent a cheaper and more statistically robust alternative to better elucidate the efficacy of GST-specific compounds against *Plasmodium* LS [101,102]. Likewise, a dose-dependent inhibition of *P. falciparum* oocyst prevalence in the midguts of *An. stephensi* was barely achieved for some compounds (e.g., CB-41 and CB-61), which was not consistent across independent experiments and was generally maintained below 50%, suggesting weak activity [35]. None of the compounds inhibited gametocyte activation, but in general, most of them were active, yet not potent, against *P. berghei* ookinetes, suggesting activity in this stage and a possible unforeseen fit to TCP-6 [35]. Although the CB series was deemed inactive against *P. falciparum* gametocytes, compounds targeting complementary redox regulators were shown to be active against stage V gametocytes [92]. In addition to the species and target–compound pairs, major differences in results might be a consequence of the type of readout, which, for the CB series, was exflagellation of microgametes and *Pb*s21 quantification in macrogametes [35], while others have used PfULG8-driven expression of luciferase in *P. falciparum* gametocytes, which is more sensitive and sex-redundant [92].

In summary, the empirical evidence described above validates GST-compound pairs as suitable for meeting TCP-1; however, fast-killing molecules against this target (e.g., for parenteral administration) have yet to be developed. Evidence of compound activity against developing LS and gametocytes is still scarce; however, evidence of GST transcription at these stages encourages efforts to meet TCP-4 and, eventually, TCP-5. Furthermore, an unforeseen activity of these compounds against ookinetes further supports target–compound pairs for transmission blocking at the mosquito stage, aligned with TCP-6. Altogether, existing findings support GST targeting in at least two stages of the life cycle, which, according to Forte and coworkers, is important for target prioritization at this level [10]. Evidence further encourages using available and developing compounds across complementary platforms (e.g., *P. berghei*-HepG2 LS, luciferase-expressing *P. falciparum* gametocytes, etc.) to validate the apparent suitability to meet TPP-1 (TCP-1 & TCP-5) and/or TPP-2 (TCP-1 & TCP-4).

#### 5.2.6. Toxicity, Novelty and Prior Information

*Plasmodium* GST is not a new target suggested for antimalarial therapies [11,47,108,109]; however, it has been deprioritized for reasons unknown to us, despite compelling evidence supporting its target-like features. Prior research has shown unique aspects of this target that may be used to galvanize the development of new molecules with improved chemistry, overcoming the existing challenges in the pipeline. Reduced selectivity of the available compounds, in particular, which in turn might lead to undesired side toxicity, is probably one of the most challenging aspects of *Plasmodium* GST targeting and the area that surely benefits the most from prior information.

As previously discussed, *Plasmodium* GST orthologs have been identified across a wide range of organisms with reduced sequence homology [11,32,37,43,44,47,48], but unfortunately, this cannot always be taken as a good correlate of selectivity. In fact, available molecules against GST have been effective in inhibiting both human and parasite enzymes through the functionally conserved G-site, which, in theory, might hinder compound selectivity when aiming to target *Plasmodium* GST. Surprisingly, a molecule such as ELA, which is often predicted to bind the G-site [38], has shown reduced toxicity in HepG2 [103], L6 myoblasts [76], and mice in vivo [75], providing an encouraging starting point for the development of a related compound series. Unfortunately, not every available compound targeting *Plasmodium* GST has the same depth of toxicity characterization, with known molecules often having their ADMET profiling done in silico [39,94,97,98]. More refined molecules, such as those in the CB series, have been shown to have no toxicity against HepG2 nor primate hepatocytes and good selectivity indexes; yet, further ADMET studies, especially those conducted in vivo are required to derive better candidate molecules from this series [35].

Despite the scarcity of toxicological studies, previous research already discussed in this review has shown that developing molecules against the less conserved and more solvent-exposed H-site might be a way forward in developing more potent and highly selective compounds against *Plasmodium* GST [94]. In fact, others have validated this assumption by showing that even a non-H-site-specific compound (e.g., ELA) can be chemically modified to target the H-site (e.g., FEA and CEA), suggesting that even simple modifications, such as the addition of hydroxyl groups, are capable of improving selectivity and potency [76]. Importantly, the improved analogs were derived from a less selective scaffold with proven antimalarial efficacy in rodent models treated intraperitoneally [75]. Despite a lack of efficiency upon oral administration, the effects observed with parenteral route administration, without visible signs of collateral toxicity, support the idea that even unoptimized molecules with drug-like properties could and should be improved by MedChem, exploring new chemical spaces that can lead to the development of newly improved compounds against this enzyme.

Based on the aforementioned evidence suggesting that improved drug-like molecules can be synthesized, we have classified *Plasmodium* GST with a high score for toxicity and a medium score for novelty/prior information, respectively.

#### 5.2.7. Assay Readiness

A wide range of tools has already been developed to screen GST-specific molecules with hypothetical antimalarial effects. Direct target validation of “hits” can be performed as the first recombinant versions of PfGST have been expressed since the early 2000s [32,41]. The recombinant production of this enzyme seems simpler than that of most proteins in *Plasmodium*; the enzyme seems to represent a more accessible alternative for direct compound testing in several laboratories around the world, where most of the already available molecules have been validated over the last decades. Alternatively, this has paved the way for expanding the synthesis of the enzyme to recombinant versions of GST orthologs in other *Plasmodium* species such as *P. vivax* [39,42,43,44] and *P. berghei* [35], allowing for the enzymatic screening of molecules with hypothetical transspecies efficacy. Moreover, recombinant versions of several human GSTs have also been produced [35,39,51,105], warranting the possibility to assess compound selectivity at the protein level. With these, biochemical assays have been extensively used to assess the inhibition of *Plasmodium* GST activity, which relies on measuring the increased absorbance resulting from the accumulation of an enzymatic by-product (spectrophotometric quantification) generated from the nucleophilic attack of GSH on CDNB promoted by the enzyme. This can be used to validate important H-site specific inhibitors, as these would compete with CDNB for the substrate binding site, consequently modifying the assay kinetics [38]. An alternative modification of the biochemical assay known as isothermal titration calorimetry (ITC), which has been used to characterize the thermodynamics behind GST-ligand interactions, is also available [105]. As previously mentioned, *Plasmodium* GST has the unique ability to exist in suspension as an inactive tetramer, with the tetramer-to-active dimer transition being mediated by GSH in solution [44,46,50], which consequently affects the accuracy of thermodynamic characterization (e.g., binding affinity and enthalpy) of ligand-protein interactions by conventional ITC. As such, a modified ITC approach has been proposed, consisting of lowering temperatures and PfGST concentrations, which favors dimer abundance with negligible levels of PfGST tetramerization. Under this optimized setup, parameters such as the binding affinity of natural ligands such as GSH could be estimated and used, for instance, as a baseline to identify stronger inhibitors of the G-site with more favorable thermodynamic characteristics [105].

In addition to expressed recombinant proteins used in biochemical assays, pipelines for antimalarial drug discovery, implemented in several laboratories around the world, rely on a wide range of phenotypic screening techniques that have not been used so far to better characterize GST-specific compounds. This is particularly important when it comes to validating TCP/TPP fitting beyond the ABS. As previously discussed, some of the available GST-specific compounds were widely tested using in vitro proliferation assays with *P. falciparum* ABS and alternative in vitro stage-specific models (e.g., *P. cynomolgi* liver stages in primate hepatocytes, microgamete exflagellation, etc.); yet, testing such compounds in more sensitive and statistically robust high-throughput models, such as those using HepG2 infected with luminescent *P. berghei* (liver stages) [101,102] and *P. falciparum* luminescent gametocytes [92], might provide better insights into the compounds’ efficiency in stages that are yet to be thoroughly validated.

Altogether, the diversity of existing assays for *Plasmodium* GST might exceed those available for other highly prioritized targets in the drug discovery pipeline, and intuitively, this should justify their use for the identification of a higher diversity of molecules and the validation of the different TPPs using the complementary phenotypic screening platforms described above. As such, we consider that, regarding the available assays, *Plasmodium* GST should have a high score on the priority list.

#### 5.2.8. Structural Information

Although not considered a prerequisite for target selection, structural information can indeed be a significant benefit in informing the rational design of potent and highly selective inhibitors. Unlike other proteins in malaria parasites, including some of the highly prioritized targets in the MalDA list (e.g., acetyl CoA synthetase, AcAS) [10], *Plasmodium* GST can be recombinantly expressed with relative simplicity, which has consequently led to a significant number of resolved crystal structures. This allowed the identification of a significant number of structural characteristics unique to this enzyme that are important for compound design and have been discussed throughout this review. Nevertheless, some less explored characteristics might have been overlooked over the years and might still represent a gamechanger for the design of new compounds against *Plasmodium* GST.

Overall, one of the most important structural observations that actually informed the synthesis of improved *Plasmodium* GST inhibitors was the absence of a Mu-loop and a more solvent-exposed H-site [37,46,49], which ended up being an important driver for compound selectivity. Compounds such as CB-27, CB-61, FEA and CEA were synthesized as a consequence of that structural knowledge [35,76,94], but no further studies were conducted to generate cocrystal structures with these compounds to empirically prove that the H-site can indeed be soaked and to determine the biochemical modifications behind this interaction. In fact, the only reported cocrystal structure of the enzyme was achieved for the G-site specific ligand GTX, providing evidence that at least the G-site of PfGST can be soaked with an inhibitor [46,49].

Some of these structures also provided additional insights for the rational design of inhibitors that might have been overlooked, including the observation of a less flexible region in the H-site pocket, which is occupied by a polyethylene glycol (PEG) molecule in the cocrystal with GTX. This molecule protrudes into the H-site of the dimer, suggesting the existence of a cavity that might be efficiently blocked with specifically designed molecules directed to both G- and H-sites [46]. Complementary observations have indicated that particular residues in that same H-site, such as those in the atypical 113–119 loop, might be important for informing sequence-directed drug design due to their essentiality to the overall enzymatic kinetics [36]. Moreover, the nearby Asn112 residue was shown to be essential for tetramerization in a phosphate- and pyrophosphate-dependent manner, supporting the idea that similar molecules with drug-like properties (e.g., created via fragment-based design [99]) could be used to enrich the inactive tetramer inside the parasite while affecting the overall GST activity [51]; a different strategy that could complement conventional catalytic inhibition. Another overlooked aspect is the existence of a non-substrate ligand-binding site (L-site) present in the monomers of the native PfGST tetramer, which seems to be naturally occupied by a 2-(N-morpholino) ethane sulfonic acid (MES) molecule, as shown by high-resolution X-ray crystallography [48]. This 10-year-old finding still lacks further experimental characterization of the impacts of L-site disturbance on the overall PfGST kinetics; however, this might be further explored for the development of new molecules against the enzyme.

Overall, structural information on *Plasmodium* GST is significantly abundant, and cocrystals with G-site ligands are available; yet simple protocols for recombinant protein production should be exploited to empirically validate that the H-site, the main driver of compound selectivity, can actually be soaked with available molecules. Nevertheless, the available structural information for *Plasmodium* GST is superior to the average target in malaria parasites and should, therefore, score high for this feature on the priority list.

## 6. Conclusions and Future Perspectives

The number of publications focusing on targeting *Plasmodium* GST for the development of antimalarials has stalled over the last decade and is aligned with the idea of a deprioritized target, characterized by the unlikeliness of fulfilling TCP requirements or by the significant abundance of related drug development programs around the world [10]. However, this is not the case for *Plasmodium* GST, and our assessment, under the criteria defined by MalDA for target candidate profiling, highlights unique aspects of the enzyme that characterize promising targets and should be considered for the development of antimalarials.

Overall, we have reinforced the unique structural and biochemical aspects of this enzyme that are important for its detoxification functions and are in line with its essentiality. Features including its dynamic oligomerization behavior and the exposed H-site can be exploited for drug targeting with important selectivity potential. Available molecules, and those that are yet to be developed, can benefit from MedChem improvement for the generation of compounds with multistage activity. Empirical evidence indicates that GST can be efficiently targeted in replicative stages of *Plasmodium* parasites, reinforced by genetic validation screens and higher transcript abundance observed during the trophozoite to schizont transition in both LS and ABS, which seems to be diminished in invasive/quiescent forms such as sporozoites, merozoites and hypnozoites, mirroring stage-specific metabolic and oxidative stress demands. Additional evidence indicates an opportunity to target transmission-related stages such as gametocytes and even ookinetes, but this also requires additional validation.

Importantly, the low prevalence of reported mutations and the high degree of GST conservation across isolates and species indicates a reduced but untested risk for resistance development, which is a highly desired characteristic in an antimalarial. Cumulatively, no cross-resistance was reported for the available molecules, overall suggesting that GST-compound pairs may also be useful to overcome resistant phenotypes. In fact, upregulation of GST activity has been linked to CQ resistance and may contribute to artemisinin (ART) tolerance by enhancing parasite resilience to oxidative damage [12], suggesting that compounds targeting GST might not work simply in monotherapy but have the potential to be included in combination therapies.

In line with this idea, a combination medicine might be required to simultaneously target other components of the parasite’s redox system; however, we cannot foresee any compensatory mechanism that could maintain redox balance upon *Plasmodium* GST inhibition [108]. In fact, it has been shown that PfGST inhibition with CEA does not lead to an upregulation of transcripts related to the antioxidant network (e.g., GR, thioredoxin reductase, glutaredoxin 1, superoxide dismutase 1), suggesting that inhibition cannot be compensated, at least via increasing transcription of those genes [76]. Nevertheless, the downregulation of these genes and their respective enzyme activity should be validated under GST conditional knockdown. Besides, it is important to highlight that there is no known enzyme encoded by the parasite genome that could also perform the addition of GSH to toxic molecules for excretion [11,47], suggesting that at least this function is unlikely to be compensated upon inhibition.

Suggested investments in compound development could take years of testing if tools were not available; however, and differently from other targets in *Plasmodium*, a wide range of tools exist and can be used for both biochemical and phenotypic screening assays. What is missing is simply the quantity and diversity of molecules developed against *Plasmodium* GST, a gap that can be easily covered by the extensive amount of structural information and bioinformatic tools available. Considering this and the relevance of this target, we cannot foresee a reason that justifies the stagnation of progress in this research area.

Our review enriches the existing literature by performing a profound target validation assessment. We bring a discussion on the functional and structural aspects of *Plasmodium* GST in the context of antimalarial drug discovery while providing a unique debate on the relevance of this enzyme beyond the ABS and its exploitation for multistage targeting. We also highlight the existence of less cited molecules that might be improved through MedChem and used to generate missing empirical data, consolidating GST validation as a target in malaria parasites. Collectively, our compiled evidence underscores the therapeutic relevance of *Plasmodium* GST, and we believe that this target should be under consideration for prioritization due to its relevance and the number of available tools [10]. We encourage further efficacy, resistance induction and safety studies to better characterize the existing compounds, as well as scaffold-based development of improved molecules using MedChem. Hopefully, GST-targeting compounds can be deemed candidates in future drug discovery initiatives, aiding in the overarching goal of eradicating malaria.

## Figures and Tables

**Table 1:** (**A**) Suitability of GST as a target under the Target Candidate Profile (TCP) and (**B**) Target Product Profile (TPP) definitions.

**(A)**			
**TCP**	**Goal**	**Definition**	**GST TCP Suitability**
TCP-1	Disease treatment and chemoprophylaxis (e.g., vulnerable populations).	Targeting ABS and all resistant strains.	Yes. Highly expressed in ABS trophozoites.
TCP-3	Anti-relapses (radical cure of recurrent malaria).	Targeting LS hypnozoites.	No. Absence of hypnozoites in *Pf*; low to null expression in *Pv*/*Pc* hypnozoites.
TCP-4	Prophylaxis (e.g., migratory populations and outbreak prevention).	Targeting developing LS.	Yes. Expression in *Pf* LS not reported, yet ortholog expression reported in *Pb*, *Pv* and *Pc* LS.
TCP-5	Transmission blockers (e.g., managing asymptomatic infections).	Targeting blood Gmt.	Maybe. Moderate expression in Gmt; yet shown to be susceptible to GR inhibitors.
TCP-6	Transmission blockers.	Mosquito stages, insecticides and endectocides.	No. Low to null expression in mosquito stages (e.g., Ooc and Spz), but evidence that Ook can be killed with GST-specific drugs.
(**B**)			
**TPP**	**Goal**	**Definition**	**GST TPP Suitability**
TPP-1	Treatment for active disease	Combination of TCP-1 with -5 or -3 to cure active disease in general population.	Yes. Compliance can be achieved by meeting TCP-1 and -5.
TPP-2	Chemoprotection	Combination of TCP-1 and -4 to prevent disease in migratory populations and outbreaks.	Yes. Compliance can be achieved by meeting TCP-1 and -4.

ABS: Asexual Blood Stage; Gmt: Gametocyte; GR: Glutathione Reductase; GST: Glutathione S-Transferase; LS: Liver Stage; Ooc: Oocyst; Ook: Ookinete; *Pb*: *P. berghei*; *Pc*: *P. cynomolgi*; *Pf*: *P. falciparum*; *Pv*: *P. vivax*; Spz: Sporozoite; TCP: Target Candidate Profile; TPP: Target Product Profile. Table adapted from Forte et al. [10].

**Table 2:** Target validation of GST according to MalDA’s list of criteria for target prioritization.

Validation Criterion	Experimental Assessment Required	Priority Ranking	Evidence of Experimental Validation?	Action (Suggested Future Steps; Missing Data in the Literature)
**Tier 1 target assessment**				
Genetic validation	Conditional KO; target vulnerability in conditional KD.	High	No conditional KO/KD reported in *Pf*.	Generation of PfGST KO/KD using conditional expression systems [95] to assess target vulnerability.
	Genome-wide saturation mutagenesis *Pf*; homologous recombination KO screens in *Pb.*	Medium	GST validated as essential for *Pf*ABS in vitro [45,55]; putative GST deemed as dispensable in *Pb* KO screens [45,82,96]; *pbgst* locus is essential for *Pb*ABS and cannot be KO [94].	NA
Chemical validation	Target–compound pair established rigorously.	High	Unavailable data on in vitro resistance induction in *Pf*ABS and available compounds followed by WGS to identify mutated target locus; unavailable data on improved drug susceptibility of transgenic parasites bearing hypothetical resistance-associated mutations.	In vitro resistance induction in *Pf*ABS pressured by sublethal concentrations of the available molecules and drug challenge transgenic parasites harboring those hypothetical mutations.
	Good correlation between enzyme and cell activity over 3 log units for a compound series.	Medium	Good correlation between the inhibition of recombinant GSTs and ABS in vitro for CB-27 [35,94], FEA and CEA [76]; correlations over 3 log units were not established due to the scarcity of synthetized series.	Synthetize new and larger compound series or upgrade existing ones with new analogs to establish meaningful correlations.
Resistance potential	Irresistible—No resistance found in selection.	High	NA	In vitro resistance induction in *Pf*ABS pressured by sublethal concentrations of the available molecules (e.g., CB-27, CB-61, FEA and CEA) [35,76,94] to evaluate the potential for resistance onset (MIR).
	MIR 8–9 and no cross-resistance with any drug in clinical use or development.	Medium	NA	
	6 < MIR< 8 and no cross-resistance with any drug in clinical use or development.	Low	*Pf*Dd2, W2, TM91c235 and C2B ABS were susceptible to CB-27 and CB-61 [35,94]; *Pf*Dd2 and K1 were susceptible to FEA and CEA [76]; an overall indication of no cross-resistance.	
	MIR ≤ 6 and an EC50 shift > 10-fold; or evidence of high-grade resistance-conferring SNPs in field isolates; or enzyme not conserved across species.	STOP	Enzyme with a single isoform conserved across sequenced species and isolates; no evidence of relevant SNPs in geographically spread field isolates.	
**Tier 2 target assessment**				
Druggability	Identification of small molecule inhibitors with drug-like physicochemical properties.	High	CB-6 and CB-19 (G-site) and CB-27 (H-site) [94]; Hydrazone CB series (CB-27 scaffold used for analogs) [35,94]; FEA and CEA (ELA analogs) [76]; knipholone [97]; compounds in IMPHY library predicted to bind GST [98], BSP [42], BA and TTMF [39] with encouraging ADMET predictions and active against recombinant enzymes.	MedChem is required to improve the lead compound series; small molecule synthesis for in vitro and in vivo efficacy tests; experimental validation of drug-like properties with ADMET studies.
	Computational analysis of the crystal structure or a high-quality homology model.	Medium	Computational analysis of the crystal structure has been used to confirm druggability and identify molecules [39,94,98,99,100]	NA
TCP/TPP fit	Must be active against two stages in the life cycle at similar concentrations or ABS with fast killing rate.	STOP/GO	Available compounds are active against ABS in vitro [35,75,76,94] and in vivo [75]; CB series is inactive against *Pb*Gmt, inconsistently active against *Pc*LS and *Pf*Ooc, but active against *Pb*Ook [35].	Use available and developing molecules in complementary assays (e.g., *Pb*-HepG2 LS [101,102], luminescent *Pf*Gmt [92], etc.) to validate TCP/TPP fit.
Toxicity	No close orthologs in humans; small molecule inhibitors against the parasite target with good selectivity indexes (vs. humans).	High	Different amino acid sequence between *Pf*/*Pv*/*Pb*GST and *Hs*GST ortholog; structural and G-site similarities but important differences in the druggable H-site [37,94]; H-site specific compounds selectively inhibit *Pf*/*Pv*/*Pb*GST but not *Hs*GST [35,39,76,94].	Poor availability of toxicological studies with available compounds (e.g., data for HepG2 [35,103], NHP hepatocytes [35], L6, A549 [76] and HeLa [104]); no toxicity reported in mice [75,76]; other in vitro and in vivo toxicological studies are required.
Novelty/prior information	Previous work has indicated issues with chemistry but potential way forward using new information/chemistry.	Medium	Evidence that selectivity and potency of new and existing molecules can be improved by chemically directing them to the H-site [76,94].	Explore chemical space considering the H-site as the major driver of selectivity when creating new compound series.
	Previous work indicated that drug-like inhibitors with in vivo activity can be generated and compound(s) in late-stage development; potential for backup compounds.	Low	Evidence for antimalarial in vivo efficacy of unoptimized drug-like inhibitors (e.g., ELA) [75] and that selectivity and potency can be improved through MedChem [76]; possibility to generated late “lead” compounds.	NA
Assay readiness	Protein expressed and biochemical assay developed for this protein or a close ortholog.	High	*Pf* [32,35,36,37,38,39,40,41,42,46,48,49,51,76,105], *Pv* [39,42,43,44], *Pb* [35] and *Hs* [35,39,51,105] GST recombinant proteins were expressed for biochemical activity assays (e.g., spectrophotometric quantification of GSH-CDNB conjugation [38], modified ITC [105]); alternative phenotypic screening assays available (e.g., *Pb*-HepG2 LS [101,102] and *Pf*Gmt [92] assays with luminescent parasites).	Apply biochemical assays to untested and new molecules; test positive “hits” against ABS, validate ABS active molecules in other stage-specific assays; re-test molecules with inconsistent stage-specific activity in complementary stage-specific assays.
	No *P. falciparum* expressed but (evidence for) assay for ortholog or reporter cell assay developed.	Medium	NA	NA
Structural information	Structure of target protein and cocrystal structures with ligands; evidence that it can be soaked.	High	*Pf*GST cocrystal structure with GTX and evidence that the G-site can be soaked [46,49].	Use available molecules (e.g., CB-27, CB-61, FEA and CEA) [35,76,94] to generate cocrystal structure preferentially targeting the H-site.
	Structure of target protein, but no complex; close ortholog with structures; potential for chimeras.	Medium	*Pf*GST crystal structure has been generated [37,46,48,49].	NA

ABS: asexual blood stage; ADMET: Absorption, Distribution, Metabolism, Excretion and Toxicity; BA: Baicalin; BSP: Bromosulfophthalein; CDNB: 1-chloro-2,4-dinitrobenzene; CEA: Coruleoellagic acid; ELA: Ellagic acid; FEA: Flavellagic acid; Gmt: Gametocyte; GSH: Reduced Glutathione; GTX: S-hexylglutathione; *Hs*: *Homo sapiens*; ITC: Isothermal titration calorimetry; KD: Knockdown; KO: Knockout; LS: liver stage; MedChem: Medicinal chemistry; MIR: Minimum Inoculum for Resistance; NA: Not applicable; NHP: Non-human primates; Ooc: oocyst; Ook: Ookinete; *Pb*: *P. berghei*; *Pf*: *P. falciparum*; *Pv*: *P. vivax*; SNP: Single nucleotide polymorphism; TTMF: 5,7,3′-Trihydroxy-6,4′,5′-trimethoxyflavone; WGS: Whole genome sequencing. Table adapted from Forte et al. [10].

## Data Availability

No new data was created or analyzed in this study. Data sharing is not applicable to this article.

## Abbreviations

The following abbreviations are used in this manuscript:

OH	Hydroxyl radical
ABS	Asexual blood stages
AcAS	Acetyl CoA synthetase
ACT	Artemisinin-based combination therapy
ADMET	Absorption, Distribution, Metabolism, Excretion and Toxicity
Ala	Alanine
Arg	Arginine
Asp	Aspartic acid
BA	Baicalin
BSP	Bromosulfophthalein
CDNB	1-chloro-2,4-dinitrobenzene
CEA	Coruleoellagic acid
CQ	Chloroquine
DNA	Deoxyribonucleic acid
EC_50_	Half maximal effective concentration
EEF	Exoerythrocytic form
ELA	Ellagic acid
ETA	Ethacrynic acid
EXP1	Exported protein 1
FACS	Fluorescence-activated cell sorting
Fe^2+^	Ferrous iron
Fe^3+^	Ferric iron
FEA	Flavellagic acid
FP	Ferri/ferro protoporphyrin IX
GFP	Green fluorescent protein
Gln	Glutamine
Glu	Glutamic acid
Gmt	Gametocyte
GR	Glutathione reductase
GSH	Reduced glutathione
GSSG	Oxidized glutathione
GST	Glutathione S-transferase
GTX	S-hexylglutathione
H_2_O_2_	Hydrogen peroxide
Hs	*Homo sapiens*
IC_50_	Half maximal inhibitory concentration
ITC	Isothermal titration calorimetry
KD	Knockdown
KO	Knockout
LC-MS/MS	Liquid chromatography tandem mass spectrometry
Leu	Leucine
LS	Liver stage
Lys	Lysine
MalDA	Malaria Drug Accelerator
MAPEG	Membrane-associated proteins in eicosanoid and glutathione metabolism
MedChem	Medicinal chemistry
MES	2-(N-morpholino) ethane sulfonic acid
Met	Methionine
MFS	Mutagenesis fitness score
MIR	Minimum Inoculum for Resistance
MIS	Mutagenesis index score
NA	Not applicable
NADPH	Nicotinamide adenine dinucleotide phosphate
NHP	Non-human primate
nsSNP	Non-synonymous single nucleotide polymorphism
O_2_	Oxygen
O_2_^-^	Superoxide
OH^-^	Hydroxide
Ooc	Oocyst
Ook	Ookinete
Pb	*Plasmodium berghei*
Pc	*Plasmodium cynomolgi*
PEG	Polyethylene glycol
Pf	*Plasmodium falciparum*
Phe	Phenylalanine
PI4K	Phosphatidylinositol-4-OH kinase
Pv	*Plasmodium vivax*
PVM	Parasitophorous vacuolar membrane
RNA	Ribonucleic acid
ROS	Reactive oxygen species
Ser	Serine
sSNP	Synonymous single nucleotide polymorphism
TCP	Target candidate profiling
Thr	Threonine
TPM	Transcripts per million
TPP	Target product profiling
TTMF	5,7,3′-Trihydroxy-6,4′,5′-trimethoxyflavone
ULG8	Upregulated in Late Gametocytes
Val	Valine
WGS	Whole genome sequencing
